# Pain in Brazilian people experiencing homelessness

**DOI:** 10.1097/PR9.0000000000000792

**Published:** 2019-12-06

**Authors:** Ariane Graça de Campos, Elivane da Silva Victor, Molly Seeley, Eliseth Ribeiro Leão

**Affiliations:** a Albert Einstein College of Health Sciences, Hospital Israelita Albert Einstein, São Paulo, Brazil; b Statistics, Instituto Israelita de Ensino e Pesquisa Albert Einstein, Hospital Israelita Albert Einstein, São Paulo, Brazil; c Institute of Global Homelessness, A Place to Call Home Program, Chicago, United States,; d Research Institute, Instituto Israelita de Ensino e Pesquisa Albert Einstein, Hospital Israelita Albert Einstein, São Paulo, Brazil

**Keywords:** Pain, Homeless, Nursing, Culturally competent health care, Access to health services

## Abstract

**Background:**

Previous studies conducted in developed countries have shown that homeless people experience a high rate of pain. In this population, pain is not only underdiagnosed but is also undertreated. In Brazil, the pain of people experiencing homelessness is unknown and this is the first study on this subject in the country.

**Objective:**

To assess and characterize the prevalence of pain in homeless people living on the streets in the center of São Paulo, Brazil, and to understand its implications to general activities according to the Brief Pain Inventory (BPI) questionnaire.

**Method:**

This is a descriptive-exploratory cross-sectional study with a quantitative approach conducted with 69 homeless people from the central region of São Paulo, Brazil. The data were captured as protected health information and analyzed with a sociodemographic form, the BPI questionnaire, the McGill Pain Questionnaire (MPQ), and the Wong–Baker Face Scale.

**Results:**

The sample consisted mainly of men who spend the night on the streets, of black and miscegenated Brazilian people, with a mean age of 51 years. This population lived an average of 12 years on the streets, presented low levels of education, and used psychoactive substances. We observed a prevalence of severe musculoskeletal pain in the spine, with intermittent activity and duration of several days. The most common self-reported worsening factor was body movement and the most common factor of improvement was use of medication, but only 15% reported pain relief. Pain greatly interfered with all BPI's Activities of Daily Living, in particular with sleep (87.2%). The MPQ was difficult for the subjects to understand. There was no evidence of an association between pain and sex. Our results suggest that the longer the time living on the street, the less pain symptoms are acknowledged by the subjects, due to the accommodation phenomenon.

## 1. Introduction

Homelessness is understood as a cause and a confounding factor to physical pain, which makes it difficult to manage pain on an ongoing basis.^41^ To date, only a few studies have addressed pain in people experiencing homelessness (PEH)^9,15,26^ and none were performed in developing countries. People experiencing homelessness are extremely vulnerable to negligence, food insecurity, consumption of untreated water, disturbed sleep, and exposure to different climatic conditions, and violence (physical, psychological, and sexual), impairing their sociocultural status^8,9,33^ and tend to experience multiple hospital admissions.^31^ For this reason, PEH may experience more physical and mental diseases than the general population.^43^ There is still a lack of information regarding pain and its impact on the life of these subjects in Brazil and other developing countries.

In this study, we adopted the definition of homeless as the individual who sleeps on the streets or in shelters and are part of a group that has common characteristics, share a culture and rules of their own that guide their living, and survive on the streets.^21^

In Brazil, homeless people are assisted by a multiprofessional health team called “Street Teams.” These professionals take care of the different problems and health needs with in loco activities, directly on the streets, and the same health team monitoring them in a specialized unit.

In our professional experience, pain is self-reported by the homeless people in general, but no standard inquiry or assessment scales are available to identify it systematically. People experiencing homelessness usually seek health care when they are in extreme pain or when the pain affects their daily activities. The aims of this study are to: (1) characterize pain in a sample of PEH according to body location, pain intensity, and improvement and worsening factors for pain; (2) characterize the impact of pain on the daily activities of PEH; (3) identify potential associations between sociodemographic variables (age, sex, educational level, and time living on the streets) and pain interference in PEH. To the best of our knowledge, this study is the first to objectively assess and analyze pain in homeless people in a city of Brazil. The results obtained here are the first step towards a better knowledge of how pain affects homeless people.

## 2. Methods

### 2.1. Study design

A descriptive-exploratory cross-sectional study.

### 2.2. Ethical aspects

The Research Ethics Committees of the Municipal Health Secretariat of São Paulo city (CAAE—Certificate of Presentation for Ethical Appreciation no 50964615.0.3001.0086) and of the Hospital Israelita Albert Einstein (CAAE no 50964615.0.0000.0071) ethically approved this study. Researchers removed individually identifiable protected health information from a data set to preserve privacy for research participants. Informed consents were obtained from all participants.

### 2.3. Participants

People experiencing homelessness were included in the study according to the following criteria: (1) were registered at a local primary care unit and were treated by the health professional street team; (2) sleeping on the street floor or in shelters, where they wander around during the day; (3) were older than 18 years; (4) at the time of the interview, to be living for at least 12 months on the street; and (5) self-reported pain in the past 3 months. Exclusion criteria were delusional speech and signs of drug or alcohol intoxication. In this study, signs of intoxication were assessed by physical examination and direct observation. Signs of abuse adopted were: sleepiness, staggering gait, red eyes and faces, slurred speech, confusion, disorientation, lack of coordination, tremors, irritability and vomiting, excitement, restlessness, mental confusion, pupil dilation, hallucinations, paranoia and verbal unrest, hyperactivity, and anxious behavior.^10^

A total of 367 participants were screened, of which 69 met the inclusion criteria, and 298 were excluded mainly due to intoxication.

### 2.4. Data collection

Data were collected from January to July 2016 in central region of São Paulo city, the largest municipality of Brazil. The participants did not receive any type of payment to participate in this study.

Most subject interviews were conducted during visits to the primary care unit in the central region of São Paulo, which is a public facility responsible for primary health care in the area. Five individuals were interviewed in shelters and 2 were approached on the streets. All interviews were conducted by a single interviewer (A.G.C.).

#### 2.4.1. Demographic information and pain assessment

According to our experience, assessing pain in PEH and gathering accurate information may be challenging tasks. Because scientific literature does not indicate the best assessment tool to use in this population, we sought to obtain as much information as possible by adopting complementary forms of pain investigation (one-dimensional and multidimensional).

Four different tools were used to collect data: a sociodemographic form on health vulnerability and pain characteristics specifically developed for this study, the Brief Pain Inventory (BPI),^7,35^ the Wong–Baker Faces Scale,^42^ and the McGill Pain Questionnaire (MPQ)^11,28^ for pain assessment.

The sociodemographic form was used to investigate age, sex, educational level, socioeconomic conditions, living conditions on the streets as well as pain characteristics such as frequency of pain, factors of improvement and worsening of pain, and unique descriptors of pain.

The BPI is a self-report questionnaire assessing pain intensity and pain interference.^7^ Total scores for pain severity and interference subscale were obtained by calculating the average of all item responses for the respective subscale (Cronbach α = 0.92 for pain severity and 0.95 for pain interference in the present cohort). Scores >4 are classified as moderate, and >7 as severe, given that persons at or above this threshold tend to have greater analgesic requirements and appraise their pain to be moderately severe.^2^

The Wong–Baker Faces Scale^42^ was used to guarantee at least the assessment of pain intensity in cases in which the application of other assessment tools was not successful.

And finally, we used the MPQ (only the part composed of pain descriptors) because it has been considered the best instrument to assess the sensitive-discriminative, affective-motivational, and cognitive-evaluative dimensions of pain. The pain index is calculated by the sum of the intensity values of the chosen descriptors, the maximum value being 78.^11,28^

### 2.5. Data analysis

Quantitative data were described by medians, and first and third quartiles due to asymmetry of measurements. Categorical variables were described as absolute frequencies and percentages. The association between categorical variables was investigated with the χ^2^ test or Fisher exact test. We used the Mann–Whitney *U* test or Kruskal–Wallis nonparametric test to compare pain between some groups (level of schooling, sex, and time living of streets). We calculated Spearman correlation coefficients (presented as ρ) to assess the association between quantitative variables. Analyses were performed using the statistical software SPPS (version 24.0) and considering a significance level of 5%.^1,17^

## 3. Results

### 3.1. Sample description

Data on sample description were obtained with the sociodemographic form on health vulnerability and pain characteristics. Of all the PEH interviewed (N = 69), most were men (65.2%) with a mean age of 51 years (range: 22–70), of black and miscegenated Brazilian people (79.7%), from the Brazilian Southeast (67%) and Northeast (39%) regions. Subjects were homeless for 12 years on average, most had some type of religion (76.8%), and completed 4 years of education or fewer (46%).

The percentage of homeless people who had some type of income within the past 30 days was 82.6%. The main origins of income were informal such as almsgiving and short-term jobs (75.4%) and government social benefit (79%). More than 50% of subjects slept on the streets, where they spent all day. Almost half of the subjects had weekly contact with their family members, mostly siblings and sons or daughters, and lived with someone else on the streets, mainly friends and partners. A significant number of individuals (84.1%) had used a psychoactive substance in the past 6 months, mainly alcohol (86.2%) and tobacco (62.1%). An average of 2.5 different types of substances per individual was identified.

All participants reported chronic pain experienced between 1 and 52 years (median of 8 years), of daily frequency, lasting from hours to days.

### 3.2. Pain profile and associations

The general pain intensity measured by the Wong–Baker Faces Pain Rating Scale was reported as severe by most participants (61.2%). The pain experience evaluated by the BPI questionnaire showed that the worst pain experienced in the past 24 hours was mainly severe (61.5%). Also, in the past 24 hours, the lighter pain was classified as severe by 30.5% of the PHE interviewed and the average pain was classified as severe by 48.4% of the participants.

During the interviews, 82.6% of the participants reported experiencing pain at the moment of which musculoskeletal pain (73.8%) was the most common. For those who had a primary complaint of headache, abdominal pain, or pelvic pain (29%), musculoskeletal pain was indicated as the secondary pain.

The number of descriptors assessed by the MPQ ranged from 0 to 20, with 50% of subjects choosing 10 or more descriptors. The MPQ pain index varied from 0 to 64, with a median of 25. We considered the descriptors used by at least 30% of subjects as the most outstanding ones to describe pain experience: sickening (44.9%), beating (43.5%), tingling (43.5%), jumping (42%), hot (40.6%), tiring (36.2%), sharp (34.8%), aching (30.4%), and unbearable (31.9%).

The rate of no choice (none) in the 20 descriptors of the MPQ subgroups ranged from 21.7% to 65.2%. Descriptors relating to the sensory dimension were the most frequently chosen (median 6, first quartile 3, third quartile 7). The spontaneous descriptors reported by subjects when trying to describe their pain were: abusive, needled, stretched, terrifying, fear of dying, constant, desperate, bruising, spreading, tight, press, hot, burn, chewed, and like body being broken.

The pain-aggravating factors mentioned included body movement (62.3%), emotional distress (36.2%), and cold weather (30.4%), among others. Regarding pain relief, the main factors cited were medication prescribed by the physicians (40.6%) and use of alcohol and drugs (23.2%). Notably, the factor “resort to religion” was not selected by subjects.

The impact of pain on daily-life activities is presented in Table 1.

**Table 1:**
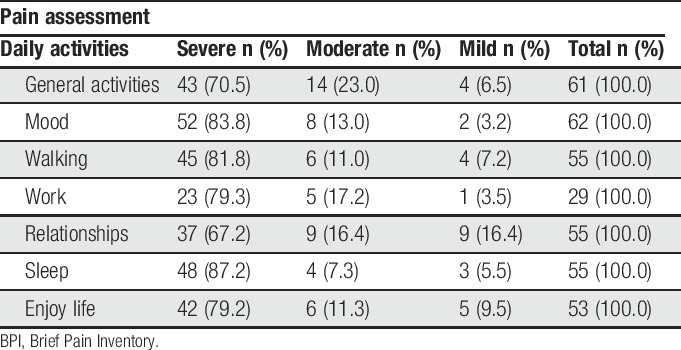
Impact of pain in daily activities (BPI questionnaire, São Paulo, Brazil, 2016).

Most of the participants (64.6%) reported not having received any type of pain treatment (only 15.3% felt pain relief) and, of those with prescribed medication, 35.4% reported having difficulties to adhere to the treatment according to the reasons presented in Table 2.

**Table 2:**
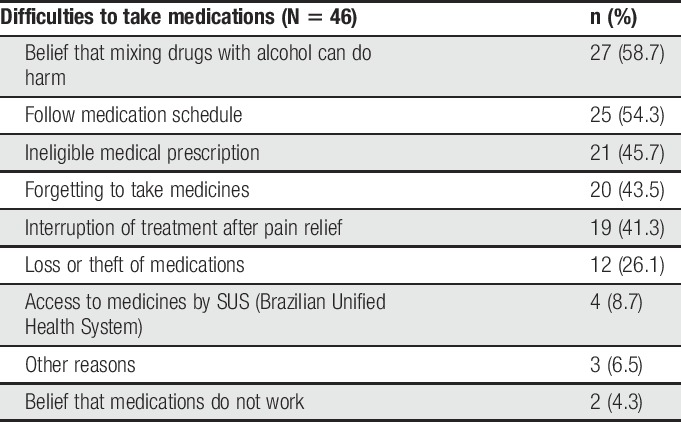
Types of difficulties reported by homeless subjects regarding analgesic medications (São Paulo, Brazil, 2016).

The MPQ did not show a strong association between pain and subject age. The highest correlation coefficient observed was in the evaluative index (0.360), suggesting that the older the subject, the greater the ability to assess pain. No relationship between sex and pain measurements was found (Table 3).

**Table 3:**
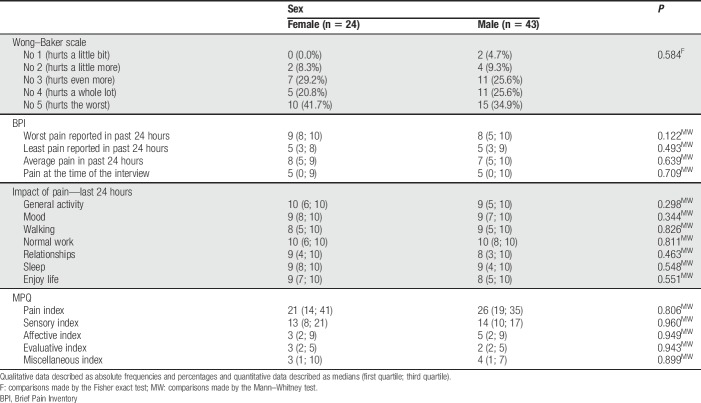
Pain measurements according to sex (Wong–Baker Scale, BPI, MPQ questionnaires, São Paulo, Brazil, 2016).

We observed an association between pain impact and education level, with lower interference on the mood (0.012), walking ability (*P* = 0.035), and relationship with other people (*P* = 0.025) of the subjects with middle-grade education (Table 4).

**Table 4:**
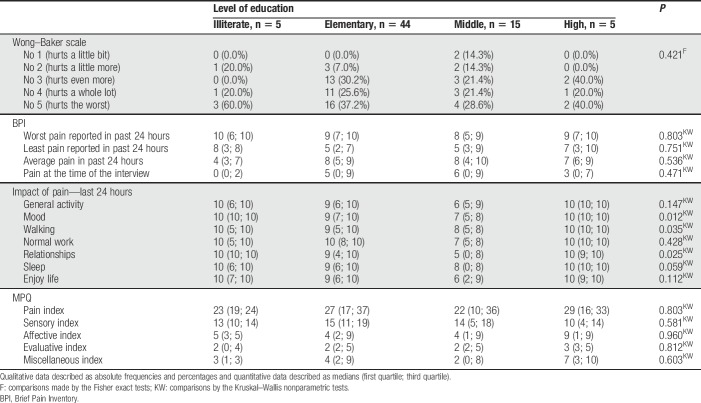
Relationship between schooling levels and pain measurements (Wong–Baker Face Scales, BPI and McGill questionnaires, São Paulo, 2016).

The association between the time spent living on the streets and pain measurement was assessed using the MPQ. Subjects were divided into 2 groups: those who lived on the streets for less than 15 years and for over 15 years. The evaluative index (*P* = 0.038) and miscellaneous index (*P* = 0.016) increased with the shortest time living on the streets.

A significant association between pain interference in sleep and time spent living on the streets (*P* = 0.039) was identified. Pain interference was higher among those who have lived on the streets for a shorter time (Table 5).

**Table 5:**
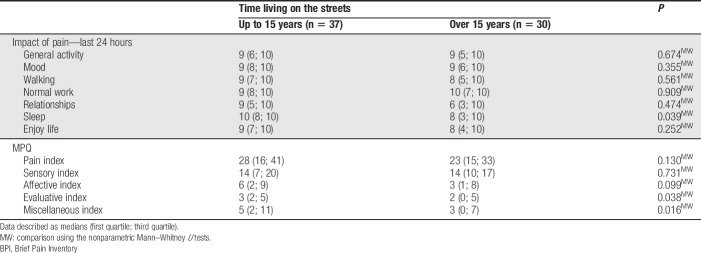
Association between duration of time living on the streets and pain measurement (BPI, São Paulo, 2016).

## 4. Discussion

This is the first study of peer-reviewed literature to describe pain characteristics in a homeless population in Brazil. Three studies have been developed in the last 10 years in developed countries^9,15,26^ and no studies of homeless people's pain in middle- or low-income countries have been found.

We present in Table 6 a summary of studies on the pain of homeless people. Although the methodologies used present differences in pain assessment, it is possible to observe some similarities: the prevalence of chronic musculoskeletal pain, severe intensity, in nonwhite mature adults.

**Table 6:**
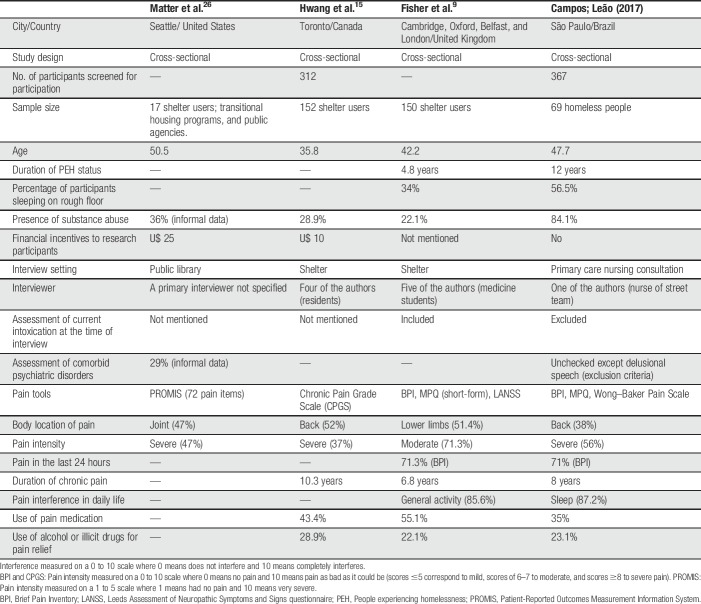
Studies on the pain of homeless people.

Our data showed that all participants had chronic pain. This finding is considerably higher than the chronic pain prevalence identified in previous studies with PEH (ranging from 47% to 59%)^9,15,26^ and more severe too (about 15%–20% higher than that reported in the literature).^9,15^ Musculoskeletal pain and headache were the most frequent types of pain.

The prevalence of chronic pain in PEH is 2 to 4 times (in the case of Saõ Paulo city) higher than that in the general population. The prevalence of musculoskeletal pain in developed countries is also relatively higher than in the general population, and those of our study curiously similar, probably due to the distribution in many other regions of the body.

Estimates suggest that 20% of adults suffer from pain globally and 10% are newly diagnosed with chronic pain each year. Consistent estimates of chronic pain prevalence in the United States range from 12% to 25%.^12^ The prevalence of low back pain is extremely common, affecting 30% to 40% of adults in the general population at any given time.^16^ A systematic review with 28 low-income and middle-income countries indicated that the prevalence of any type of chronic pain was 33% in the general adult population.^18,19^

The number of homeless people worldwide is estimated to be between 100 million and 1 billion, depending on how we count them and the definition used.^36,44^ In 5 different high-income countries, the experiences with homelessness by nation were: 280.6 million of people (United States), 10.3 (Belgium), 60.1 (UK), 83.3 (Germany), and 58.0 (Italy). However, little is known about the causes of homelessness or the characteristics of homeless people in developing countries.^37^

According to the last Brazilian census in 2015, there were 101,854 PEH, of those 15,905 were in the city of São Paulo (higher than that of Belgium), where the study was conducted.^34^ If the prevalence of chronic pain in the city of São Paulo is 4 times higher than that in developed countries, and if we take as an example, Manila (Philippines), which has the largest homeless population of any city in the world—3.1 million,^23^ it is possible to estimate a significant number of people in developing countries, and even more in low-income countries whose pain can be severe and unknown.

Our study seeks a way to indicate that it is necessary to give a voice to this population whose pain is even scientifically neglected, besides offering the first parameters on the pain of homeless people outside developed countries.

Pain location and intensity in Brazilian homeless people may be related to sleeping rough on the streets, on cold or wet floor. Factors such as carrying heavy objects and standing up for a long time were associated with worsening pain. Risk factors for low back pain included age, sex, body mass index, muscle imbalance, muscular strength, socioeconomic conditions, and other comorbidities.^27^ Several factors commonly observed in PEH that contribute to chronic pain include psychosocial conditions, substance abuse, smoking, genetic factors, financial and marital status, postural habit, educational level, and previous and/or current heavy work.^5^

According to the Brazilian National Census, PEH frequently have an occupation related to civil construction (27.2%), as domestic and mechanical work.^3,4^

These differences in prevalence are possibly related to the different study characteristics, such as design, place for data collection, availability of gratification for participation in the research, and tools used for pain assessment.

Studies have performed data collection in shelters users.^9,15,26^ Our study showed that a large percentage of subjects in our sample spent the day (71%) and night (56.5%) on the streets, higher than what was observed in a study in the United Kingdom that included the majority of participants from the night shelters (63.3%).^9^

The most-cited factors for pain relief were analgesic medications (40.6%), but some subjects reported difficulty in using those (35.4%) due to alcohol and drugs consumption. These findings, however, are lower than those found in Canada, where Canadian PEH reported the use of nonprescription medications (48%), street drugs (46%), prescribed medications (43%), and alcohol (29%) to treat their pain. The most common self-reported barrier to pain management is the inability to afford prescribed medications,^15^ which we also observed in our study.

Regarding pain assessment with the MPQ questionnaire, the groups of descriptors most cited were the sensorial-discriminative, which corroborates findings from the literature.^9^ It was not the objective of this study to evaluate the tools for pain assessment among them, but some aspects have caught our attention. Of the 20 subgroups of descriptors for pain, 12 had the first descriptor chosen (a mildest descriptor). This is contradictory when we consider that most people reported severe pain (measured by Wong–Baker Scale and BPI), which suggests that these subjects may have difficulty in understanding the descriptors.

Measuring pain among PEH is challenging. According to another study on pain assessment, some subjects have difficulty in understanding the MPQ descriptors due to the low level of education, or cognitive or verbal impairment.^11^ In our study, the words had to be repeated many times and therefore of the 20 subgroups, 12 had the first descriptor selected, demonstrating the fragility and inadequacy of the MPQ for this population. This observation reinforced the importance of having used the other pain assessment scales in this study. The literature suggests that homeless subjects should be included in the development of assessment tools, validation studies, and other clinical research to ensure that results are relevant and applicable to their atypical life conditions.^26^

Our results also showed a relevant impact of pain on daily activities compromising mainly sleep (87.2%), mood (83.8%), and walking (81.8%). High sleep impairment may be related to the fact that most subjects slept on rough floor daily or were exposed to the weather, as well as the possibility of the occurrence of physical violence and use of psychoactive substances.

The high impact of pain on walking activities in homeless people is worrying, considering the fact that in the central region of the city, where this research took place, there is only one shelter and no cohabitation centers, which are places for personal hygiene or public feeding places. This requires that subjects walk long distances to have access to these services in other shelters or improvised and remote places.

According to a study conducted in the United Kingdom, most PEH with pain reported general activity as the most affected by pain. Relationships seem to be the activity least affected by pain for over half of participants (54.1%).^9^

In this study, relationships were also the least affected activity for 67.2% of subjects, probably due to the fact that the separation between PEH and their families is a recurring situation. The relational arrangements that emerge on the streets seem to be less affected due to their fragility or to bonds of solidarity.

Sex, age, and level of education have been identified as factors that interfere in pain experience.^22,39^ In our study, we found no evidence of association between pain and sex, in disagreement with studies in general on sex and pain.^25,29,32^ This finding allows the reflection on how much the sex issue can be suppressed to make street life possible; pain expression in a woman must be sublimated or denied to survive on this predominantly male universe.

The relationship between the subject age and pain was also analyzed and we found a mild association, suggesting that the older the subject, the greater the ability to evaluate pain (MPQ). Previous studies have reported that the elderly have greater control over pain and a good adaptation to chronic pain, and mistakenly suggest that pain is part of the aging process.^11,20^

We observed an association between pain and level of education, suggesting that the higher the level of education, the greater the ability to assess pain. Also, we observed a predominance of low education level. One of the largest prevalence studies on pain in low- and middle-income countries conducted by researchers in the city of São Paulo showed 28% of people with chronic pain, with association between illiteracy with more prevalent pain.^24^

There was also a great impact of pain (BPI) on mood, walking, and relationships with other people, with greater interference among the illiterate and those with higher education. Although a greater cognitive repertoire translates into a higher capacity for analysis and decision, researchers point that low schooling increases pain tolerance.^6,30^

To the best of our knowledge, there are no studies relating walking and educational level; only studies in the general population showing that lower prevalence of chronic pain is associated with a high educational level, regular practice of physical activity, paid work, and good socioeconomic status.^38^

We observed that subjects who lived on the streets for more than 15 years presented reduced evaluative indices (MPQ results). The same was observed for the impact of pain on sleep, which was less affected with longer exposure to street life. A longer time living on the streets seems to result in greater accommodation and pain resilience.^13^ Therefore, pain will only be noticed when it interferes significantly with daily activities or when it is unbearable.

Studies about pain in PEH reveal the researchers' difficulties in accessing this population and including them in the research.^9,15,26^ These studies clearly indicate difficulties with inclusion criteria, unwillingness of participants to answer questions, which implies poorly representative samples, difficulties in understanding questionnaires due to substance abuse (including for pain relief), and the evaluation of other psychiatric disorders. This study also faced similar difficulties.

There are 2 major limitations that could be addressed in future research. First, this study focused on a small sample of PEH in a country of continental dimensions, which may bias sample population. Second, a large number of subjects were excluded due to signs of intoxication by psychoactive substances or delusional speech. Concomitant psychiatric disorders are a relevant factor in this population^34,40^ and were not considered in this study sample. Therefore, the implication of pain in such subjects is unknown and requires evaluation in future studies as well as larger sample to increase internal validity.

Recruitment difficulties also generate data bias in the analyzed literature. In 2 studies,^15,26^ shelter participants received financial incentive for completing the interview, and this incentive may have attracted some participants who did not legitimately meet study inclusion criteria. In Brazil, this procedure is prohibited by the legislation that regulates research with human beings.

It is noteworthy that the nurse who has worked with this population for 8 years (in the street team) was a fundamental factor in obtaining the data, due to a trust relationship between the participant and this professional. She was also responsible for nursing consultation in the primary care unit, where the data were collected, which may also have caused some bias, but it was the only possible strategy for conducting data collection, given that street noise and lack of privacy were major impediments to field assessment.

All these difficulties generated important limitations and should be considered in future research to expand this theme in this vulnerable population.

This study showed that intense and chronic pain is present in PEH and that it has an important impact on their daily activities. Pain control in the studied sample was also inefficient. We hope that these data can sensitize health professionals to develop actions focused on pain management specifically aimed at this population and to a consequent reduction of the suffering to which they are exposed.

Training for front-line staff on the link between pain and homelessness is needed to understand the impact it has on the lives of people, how to support, and access support. There is a need for pain specialists to have a better understanding of homelessness and the often complex needs their patients may be dealing with and the impact this may have on their ability to engage in treatment.^14^

Knowing the pain characteristics of PEH is helpful to decide on what pain assessment tools should be used in the future.

## Disclosures

Part of the results of this study was presented at the 17th IASP World Congress on Pain 2018, Boston, MA.
